# Authorship inequalities in global health research: the IeDEA Southern Africa collaboration

**DOI:** 10.1136/bmjgh-2023-013316

**Published:** 2023-12-15

**Authors:** Veronika W Skrivankova, Stefanie Hossmann, Morna Cornell, Marie Ballif, Carole Dupont, Jacqueline Huwa, Konstantinos Seintaridis, Thokozani Kalua, Gilles Wandeler, Reshma Kassanjee, Andreas D Haas, Karl-Gunter Technau, Lukas Fenner, Nicola Low, Mary-Ann Davies, Matthias Egger

**Affiliations:** 1 Institute of Social and Preventive Medicine (ISPM), Universität Bern, Bern, Switzerland; 2 Centre for Infectious Disease Epidemiology & Research, School of Public Health, Faculty of Health Sciences, University of Cape Town, Cape Town, South Africa; 3 Department of Infectious Diseases, Bern University Hospital, Inselspital, University of Bern, Bern, Switzerland; 4 Lighthouse Trust, Lilongwe, Malawi; 5 Department of HIV and AIDS, Malawi Ministry of Health, Lilongwe, Malawi; 6 Department of Paediatrics and Child Health, School of Clinical Medicine, Faculty of Health Sciences, University of the Witwatersrand, Johannesburg, South Africa; 7 Population Health Sciences, Bristol Medical School, University of Bristol, Bristol, UK

**Keywords:** Health policies and all other topics

## Abstract

**Background:**

The International epidemiology Databases to Evaluate AIDS conducts research in several regions, including in Southern Africa. We assessed authorship inequalities for the Southern African region, which is led by South African and Swiss investigators.

**Methods:**

We analysed authorships of publications from 2007 to 2020 by gender, country income group, time and citation impact. We used 2020 World Bank categories to define income groups and the relative citation ratio (RCR) to assess citation impact. Authorship parasitism was defined as articles without authors from the countries where the study was conducted. A regression model examined the probability of different authorship positions.

**Results:**

We included 313 articles. Of the 1064 contributing authors, 547 (51.4%) were women, and 223 (21.0%) were from 32 low-income/lower middle-income countries (LLMICs), 269 (25.3%) were from 13 upper middle-income countries and 572 (53.8%) were from 25 high-income countries (HICs). Most articles (150/157, 95.5%) reporting data from Southern Africa included authors from all participating countries. Women were more likely to be the first author than men (OR 1.74; 95% CI 1.06 to 2.83) but less likely to be last authors (OR 0.63; 95% CI 0.40 to 0.99). Compared with HIC, LLMIC authors were less likely to publish as first (OR 0.21; 95% CI 0.11 to 0.41) or last author (OR 0.20; 95% CI 0.09 to 0.42). The proportion of women and LLMIC first and last authors increased over time. The RCR tended to be higher, indicating greater impact, if first or last authors were from HIC (p=0.06).

**Conclusions:**

This analysis of a global health collaboration co-led by South African and Swiss investigators showed little evidence of authorship parasitism. There were stark inequalities in authorship position, with women occupying more first and men more last author positions and researchers from LLMIC being ‘stuck in the middle’ on the byline. Global health research collaborations should monitor, analyse and address authorship inequalities.

**WHAT IS ALREADY KNOWN ON THIS TOPIC**

- Studies have shown that women and authors from low-income and middle-income countries are under-represented in the first and last authorship positions compared with their colleagues from high-income countries (HICs).
- Authorship parasitism, defined as articles without authors from the countries where the study was conducted, has been shown to be common for some countries.

**WHAT THIS STUDY ADDS**

- This study analysed the publication output of the International Databases to Evaluate AIDS (IeDEA) in Southern Africa, which is co-led by researchers from South Africa and Switzerland, with sites in Lesotho, Malawi, Mozambique, South Africa, Zambia and Zimbabwe.
- In contrast to previous studies, women were more likely to be the first author than men. However, the proportion of female authorships declined when moving from first to last authorship. Authors from HICs tended to occupy the first and last positions. All analyses of the Southern African region of IeDEA included authors from participating countries.

**HOW THIS STUDY MIGHT AFFECT RESEARCH, PRACTICE OR POLICY**

- Global health research collaborations should include a process of review and reflection on equity in roles, responsibilities and authorship. This in-depth analysis of authorships will inform measures to increase participation in IeDEA Southern Africa, and may serve as an example for other collaborations.

## Introduction

Global health has been defined as an area for study, research and practice that aims to improve health and achieve equity in health for all people within frameworks of international co-operation and global solidarity.^1^ The number of articles published in global health journals increased sharply in recent decades, primarily due to the emergence of new journals.^2^ Research collaborations involving investigators from the North and South play a critical role in generating essential data to tackle the burden of disease and promote global health. These collaborations not only contribute to scientific progress but can also help build capacity, foster mutual learning and promote equitable access to resources.^3–5^

Academics participating in global health partnerships also pursue other goals, such as their recognition as experts in the field, promotion and job security and the acquisition of resources and prestige for their institutions. Shiffman argued that imbalances in financial and social resources, capacity and skills and legitimacy may lead to a ‘field of unequal power relations’ among those based in the countries from different income groups, between historically advantaged and disadvantaged and between men and women.^6^ Authorship inequalities are a manifestation of such power relationships.

Authorship parasitism is defined as articles without authors from the countries where the study was conducted.^7^ Rees *et al* found that such parasitism was very common for some sub-Saharan African countries (eg, in over 70% of articles reporting research from Somalia or Eritrea), but less common for other countries, for example, South Africa (7.1% of articles).^8^ Merriman *et al* found that both women and men from low-income and middle-income countries were under-represented as first and last authors, but women from low-income and middle-income countries were particularly uncommon in these positions compared with their counterparts from high-income countries (HICs).^9^

The International epidemiology Databases to Evaluate AIDS (IeDEA) is a global collaboration of researchers from low-income countries, middle-income countries and HIC with seven regional data centres. IeDEA was established in 2007 to examine the delivery and outcome of antiretroviral therapy (ART) and to study HIV-related comorbidities and coinfections, including hepatitis, tuberculosis and non-communicable diseases.^10 11^ We examined authorships and authorship positions, time trends and citation impact for articles involving the Southern African region of IeDEA (IeDEA-SA), by gender and country income level.

## Methods

### The International epidemiology Databases to Evaluate AIDS (IeDEA)

In 2006, the National Institute of Allergy and Infectious Diseases invited applications for consortia from regional centres collecting longitudinal data on people living with HIV on ART to answer questions that cannot be answered by individual cohorts. IeDEA began its work in 2007 and covers seven geographic regions: four in sub-Saharan Africa (West Africa, Central Africa, East Africa and Southern Africa), the Caribbean, Central and South America; Asia-Pacific and North America.^10 11^ The investigators in the regional data centres collaborate with local site investigators who are mostly clinicians. The project is funded in 5-year cycles, with the current funding cycle ending in 2026. All investigators can submit proposals for analyses.

The IeDEA-SA consortium has two co-principal investigators (M-AD, woman, and ME, man), with data centres at the University of Cape Town, South Africa, and the University of Bern, Switzerland. The database includes over 1 million people living with HIV enrolled in 17 treatment programmes in six countries: Lesotho, Malawi, Mozambique, South Africa, Zambia and Zimbabwe.^12^ IeDEA-SA authorship guidelines mandate that collaborators providing data have the opportunity to contribute to manuscripts, with the aim of including authors from all countries represented in an analysis. For multiregional analyses, all regions contributing data, but not all countries, must be represented. Authors must meet the criteria of the International Council of Medical Journal Editors (ICMJE).^13^ Since 2007, the investigators in the Southern Africa region have published regional studies and participated in multiregional projects that include data from other, or all, of the seven IeDEA regions. IeDEA investigators also published methodological work, systematic reviews and commentaries.

### Inclusion criteria and data sources

Our inclusion criteria for publications were publications from 2007 to 2020 and an acknowledgement of the main funding source National Institute of Health Cooperative Agreement AI069924) in the PubMed database. We downloaded the publication list from the IeDEA-SA website^12^ on 26 April 2021. We extracted the following metadata from the Scopus^14^ database entry for each article, using its PubMed identifier (PMID): title, first and last names of each author, country of affiliations of each author and date of publication. We excluded papers with group authorship and no writing committee. For the publications based on data from the Southern African region, VWS and AH independently determined whether all countries contributing data were represented on the author list. Discrepancies were resolved in discussion with M-AD and ME.

### Data management

We harmonised the author names by removing accents, capital letters, apostrophes and dots from first names and last names and used the Stata command ‘strgroup’ to correct typographic errors.^15^ We dealt with missing values in first names by replacing the initial with the full first name available from other entries of authors with the same last name and matching initial. We recovered missing affiliations by imputing the data using values from another publication of the same author, conducting a literature search of the authors or contacting the corresponding authors. In the final dataset, each line represented an authorship, with a unique identifier for the author, PMID for the article and author-level and article-level information.

### Definitions

We determined authors’ gender as woman or man, using two classifiers of first names. The R package ‘gender’^16^ uses the US Social Security Administration dataset of first names from the US census. The GenderChecker.com database^17^ is compiled from the UK census data. If the two sources did not agree, we contacted the corresponding author to ask about the author’s gender. We did not consider non-binary gender identities or changes in gender.

For authors who listed multiple affiliations, we defined the main affiliation as the place where the author spent most of their time when the article was published. If necessary, we contacted authors to confirm main affiliations. We assigned the income category to the country of each author’s main affiliation using the World Bank 2020 country classification.^18^ We generated three groups: lower-income countries (LICs) (low-income and lower middle-income countries, LLMICs), upper middle-income countries (UMICs) and HICs. We combined the LLMICs into one group due to low numbers of authors in each group.

We recorded three measures of impact and visibility. First, we submitted the list of PMIDs to the iCite website^19^ of the NIH Office of Portfolio Analysis and obtained the relative citation ratio (RCR, as of March 2022). The RCR uses the co-citation network to normalise the number of citations an article has received to its field.^20^ A ratio of 1 means that the article was cited as frequently as the comparison group of NIH-supported articles from the network. Second, we obtained from iCite the number of papers that had been cited in a clinical document, for example, in guidelines. Third, we assessed the open access status of articles using the Unpaywall website.^21^

### Statistical analysis

We standardised authorship positions by converting them into percentiles. First authorships corresponded to the zero percentile and last authorships corresponded to the 100th percentile. Other authorship positions were equidistantly scaled between 0 and 100. We classified single-author articles as first authorships. For visual comparison, we plotted relative proportions of each comparison group as a function of the standardised authorship position, together with a fitted regression line for gender and cubic spline curves for affiliation. The regression line and cubic spline curves were weighted by the number of authorships at each standardised position.

We assessed gender and country income differences in authorship position (first and last author vs middle authorship) using a generalised multinomial regression model. The outcome was the standardised authorship position, grouped into five categories: 0 percentile (first authorship), 1–33 percentile, 34–66 percentile, 67–99 percentile and 100 (last authorship), with 34–66 percentile as the reference category. The model included author-level random intercepts to account for correlation between authorships by the same author. We examined time trends and tested the interaction between the year of publication and gender. Results are shown as crude or adjusted ORs with 95% CIs. Finally, we compared the median RCR for first and last authorships, by gender and country income. All analyses were performed using R (V.4.1.1, R Foundation for Statistical Computing, Vienna, Austria).

To examine whether results differed by type of publication, we repeated analyses separately for regional analyses, multiregional analyses and other publications that acknowledged IeDEA-SA funding.

## Results

We downloaded 320 articles published from 2007 to 2020. Seven publications were excluded because they had group authorship without a writing committee or did not acknowledge the grant. The remaining 313 articles had 1064 authors and included a total of 3421 authorships. The median number of authors per article was 10 (IQR 7–14, range 1–75). Authors’ gender was identified using the databases of names (2744; 80.2%), by requests to the corresponding author (661; 19.3%) or through internet searches (16; 0.5%). Missing affiliations were recovered using information from other database entries (203; 5.9%) or from literature and internet searches (164; 4.8%). The 313 articles were published in 58 different journals (see online supplemental appendix p 2-3 for list of articles and p 4-5 for list of journals).

10.1136/bmjgh-2023-013316.supp1 Supplementary data

The publications reported analyses of data from the Southern African IeDEA region (157, 50.2%), multiregional analyses (95, 30.3%) or were other items acknowledging the NIH award (61, 19.5%). All multiregional analyses included authors representing the regions involved. All 157 regional articles led by IeDEA-SA included authors from at least one participating country and all but seven articles (4.5%) included authors from all the countries contributing data. The countries contributing data but not represented on the byline of the seven articles were Lesotho, Mozambique and Malawi. Authorships on the other publications (methods papers, systematic reviews or commentaries) were dominated by authors from South Africa and Switzerland.

### Gender and country income level

Among the 313 papers, 18 (5.8%) had no woman and 10 (3.2%) had no man as author. Further, 175 (55.9%) items had no author from an LIC, 57 (18.2%) had no author from a UMIC and 34 (10.9%) had no author from a HIC. In total, 22 (7.0%) papers had authors from HICs only. The latter were mainly commentaries and reviews, none of them analysed IeDEA-SA data.

Among the 1064 authors, there were more women (547, 51.4%) than men (517, 48.6%) (table 1). Female investigators authored on average 2.7 (SD 6.2, range 1–99) articles, compared with 3.8 (SD 9.9, range 1–158) for men. Women were more likely to be first authors and men were more likely to be last authors. Authors’ main affiliations were in 32 LICs, 13 UMICs and 25 HICs. HICs contributed the largest proportions of both authors and authorships, followed by UMICs and LICs. The differences were most pronounced for first and last authorship, with only 33/313 (10.5%) of authorships from LICs. There were similar numbers of published articles per author from HICs (mean 3.6, SD 10) and UMICs (mean 3.5, SD 7.2), but fewer from authors from LICs (mean 1.9, SD 2.5).

**Table 1:** Characteristics of authors and authorships of 313 articles published by the International epidemiology Databases to Evaluate AIDS in Southern Africa, 2007–2020

	Authors N (%)	Authorships N (%)	No. of articles per author Mean (SD)	First authorships N (%)	Last authorships N (%)	First and/or last authorships N (%)
Total	1064	3421	3.2 (8.2)	313	311*	313
Gender						
Woman	547 (51.4%)	1480 (43.3%)	2.7 (6.2)	173 (55.3%)	104 (33.4%)	213 (68.1%)
Man	517 (48.6%)	1941 (56.7%)	3.8 (9.9)	140 (44.7%)	207 (66.6%)	248 (79.2%)
Country of affiliation						
High income	557 (52.3%)	1679 (49.1%)	3.6 (10)	184 (58.8%)	190 (61.1%)	228 (72.8%)
Woman	278 (49.9%)	731 (43.5%)	3.1 (7.9)	104 (56.5%)	67 (35.3%)	141 (61.8%)
Man	279 (50.1%)	948 (56.5%)	4.1 (12)	80 (43.5%)	123 (64.7%)	157 (68.9%)
Upper middle income	275 (25.8%)	1187 (34.7%)	3.5 (7.2)	100 (31.9%)	112 (36%)	133 (42.5%)
Woman	178 (64.7%)	585 (49.3%)	2.7 (4.4)	63 (63.0%)	36 (32.1%)	83 (62.4%)
Man	97 (35.3%)	602 (50.7%)	4.9 (11)	37 (37.0%)	76 (67.9%)	93 (69.9%)
Lower income†	232 (21.8%)	555 (16.2%)	1.9 (2.5)	29 (9.3%)	9 (2.9%)	33 (10.5%)
Woman	91 (39.2%)	164 (29.5%)	1.5 (1.1)	6 (20.7%)	1 (11.1%)	7 (21.2%)
Man	141 (60.8%)	391 (70.5%)	2.2 (3.1)	23 (79.3%)	8 (88.9%)	29 (87.9%)

For authors who listed multiple affiliations, we defined the main affiliation as the place where the author spent most of their time when the article was published. The percentage of women/men within an affiliation income category was calculated from total in the corresponding income category. World Bank high-income countries (2020): Australia, Austria, Belgium, Canada, Chile, Denmark, Estonia, France, Germany, Greece, Italy, Latvia, Lithuania, Netherlands, New Zealand, Portugal, Saudi Arabia, Singapore, South Korea, Spain, Sweden, Switzerland, Taiwan, the UK and the USA. World Bank upper middle-income countries (2020): Argentina, Belarus, Botswana, Brazil, China, Iran, Iraq, Malaysia, Mexico, Peru, Romania, South Africa and Thailand. World Bank lower-income countries (LICs) (2020): Angola, Benin, Burkina Faso, Burundi, Cambodia, Cameroon, Congo, Côte d'Ivoire, Democratic Republic Congo, Ethiopia, Ghana, Honduras, India, Indonesia, Kenya, Lesotho, Malawi, Mali, Moldova, Morocco, Mozambique, Nepal, Nigeria, Rwanda, Senegal, Tanzania, Togo, Tunisia, Uganda, Vietnam, Zambia and Zimbabwe.

*Authorships from two single-author publications were treated as first authorships.

†LICs include World Bank lower middle-income countries and low-income countries. There were 131 and 101 authors and 327 and 228 authorships from lower middle-income and low-income countries, respectively.

### Standardised authorship position

**Figure 1:**
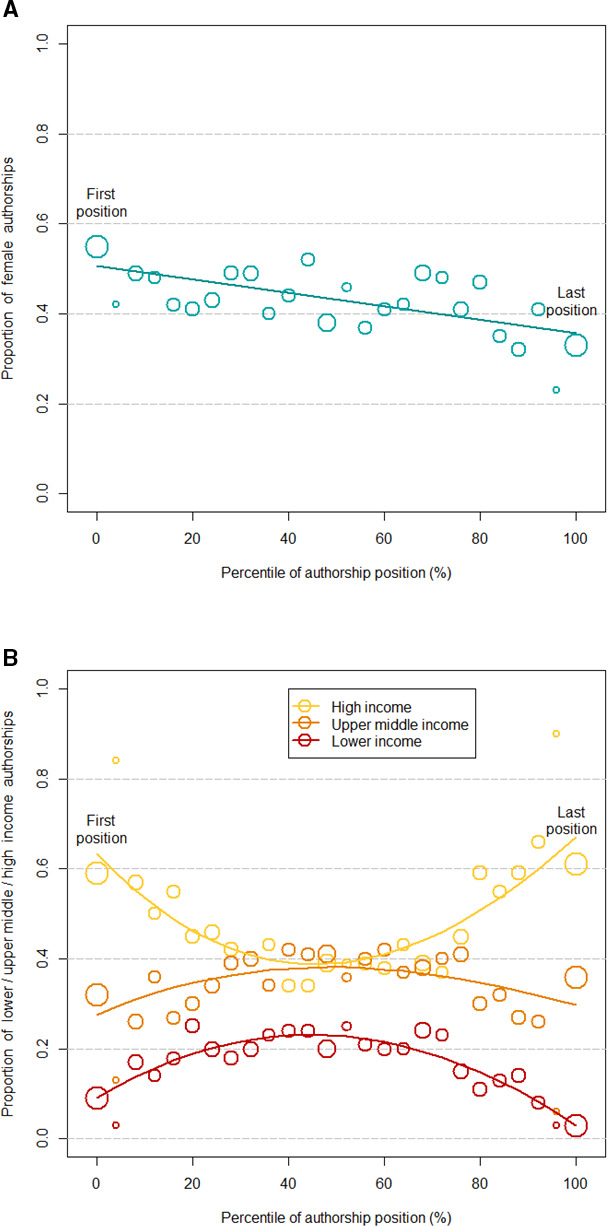
Proportion of female authorships across the range of standardised authorship position, with a weighted linear regression line (A) and proportion of authorships by country income level with weighted cubic splines (B). The size of the circles is proportional to the number of authorships in each position. Lower-income countries include World Bank lower middle-income countries and low-income countries.

Figure 1 shows the proportion of authorships from women and the proportions from HICs, UMICs and LICs across the standardised authorships positions. The proportion of female authorships declined linearly when moving from first to last authorship position (A). Affiliations from HICs dominated in the first and last positions, followed by upper middle-income and lower-income affiliations (B). The fitted cubic splines showed a U-shaped curve with higher proportions of high-income authorships in the first and last positions as compared with the authorships in the middle and inverted U-shaped relationships for the UMICs and LICs.

In the multinomial regression analyses adjusted for gender and country income level, these trends were confirmed (table 2). Women were more likely to be first authors (adjusted OR (aOR) 1.78, 95% CI 1.09 to 2.92), but less likely to be last authors (aOR 0.59, 95% CI 0.37 to 0.94) than men. Compared with authors from HICs, authors with main affiliations in LICs were less likely to be first authors (aOR 0.30, 95% CI 0.16 to 0.57) and last authors (aOR 0.15, 95% CI 0.06 to 0.34). The odds of being a first or last author were also lower for authors from UMICs than for authors from HICs (table 2).

**Table 2:** Associations of gender and country of affiliation with authorship position

Comparison/authorship position	Univariable models		Multivariable model	
	OR (95% CI)	P value	aOR (95% CI)	P value
Gender: woman versus man				
0 percentile (first)	1.90 (1.15 to 3.13)	0.01	1.78 (1.09 to 2.92)	0.02
1–33	1.11 (0.69 to 1.78)	0.68	1.07 (0.66 to 1.73)	0.78
34–66	1		1	
67–99	1.00 (0.66 to 1.52)	0.99	0.96 (0.64 to 1.46)	0.86
100 percentile (last)	0.69 (0.44 to 1.09)	0.11	0.59 (0.37 to 0.94)	0.02
Country of affiliation				
Lower versus high income				
0 percentile (first)	0.27 (0.14 to 0.52)	<0.001	0.30 (0.16 to 0.57)	<0.001
1–33	0.60 (0.36 to 1.01)	0.06	0.61 (0.36 to 1.03)	0.06
34–66	1		1	
67–99	0.61 (0.39 to 0.98)	0.04	0.61 (0.38 to 0.98)	0.04
100 percentile (last)	0.16 (0.07 to 0.37)	<0.001	0.15 (0.06 to 0.34)	<0.001
Upper middle versus high income				
0 percentile (first)	0.53 (0.31 to 0.92)	0.02	0.50 (0.29 to 0.86)	0.01
1–33	0.74 (0.44 to 1.24)	0.25	0.73 (0.43 to 1.24)	0.25
34–66	1		1	
67–99	0.79 (0.50 to 1.25)	0.31	0.79 (0.50 to 1.26)	0.33
100 percentile (last)	0.67 (0.42 to 1.08)	0.10	0.69 (0.43 to 1.11)	0.12

Results from generalised multinomial regression model with random intercept for authors. Multivariable model is adjusted for gender and income level of the country of affiliation. Lower-income countries include World Bank lower middle-income countries and low-income countries.

aOR, adjusted OR.

### Time trends

**Figure 2:**
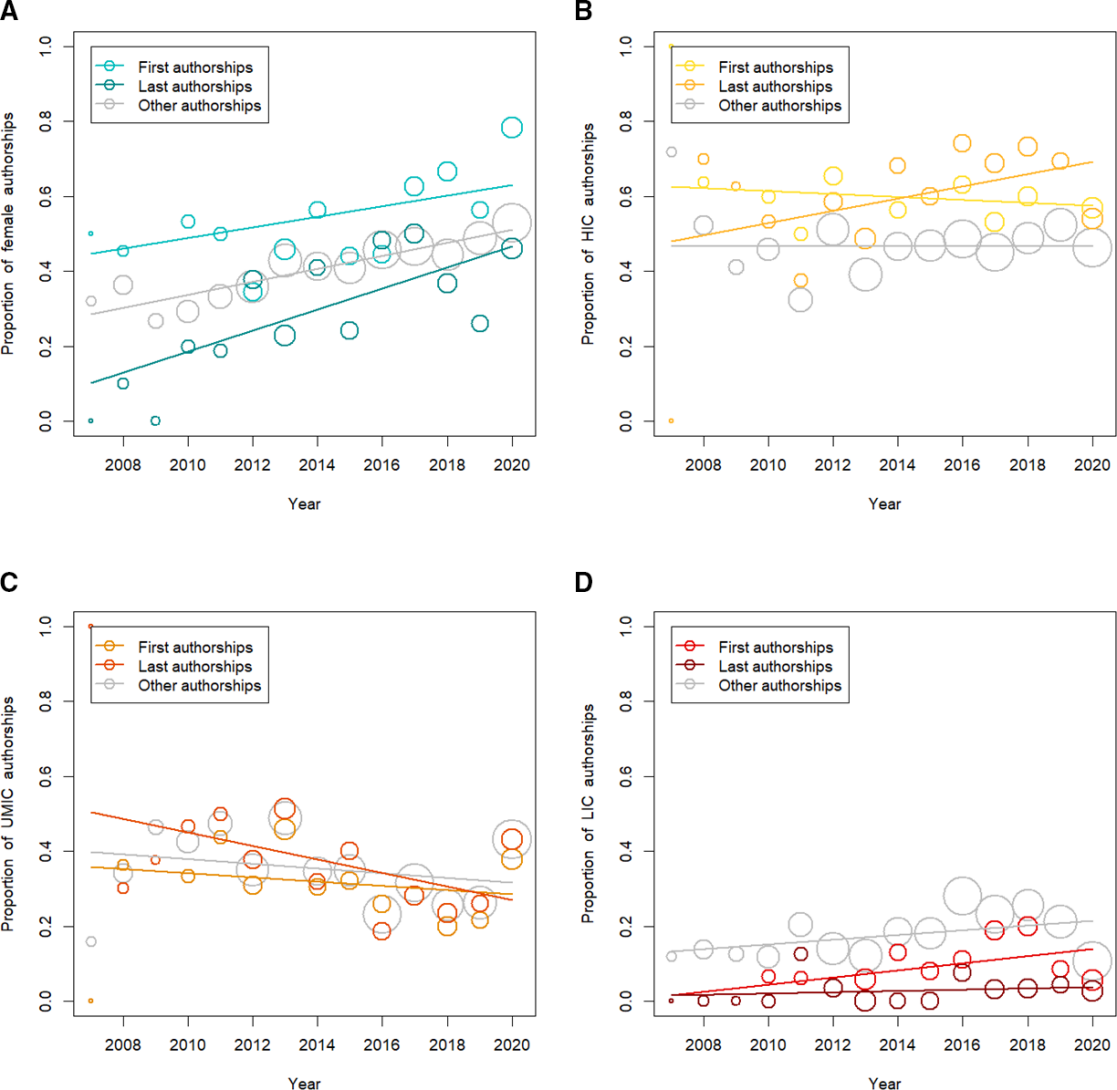
Proportions of first, last and other authorships over time for female authors (A), authors from high-income countries (HICs) (B), upper middle-income countries (UMICs) (C) and lower-income countries (LICs) (D). The size of the circles is proportional to the number of authorships. Lower-income countries include World Bank lower middle-income countries and low-income countries.

From 2007 to 2020, the proportion of women who were either first or last authors increased (figure 2A). The same increases were observed for the proportion of authors from low-income countries and HICs (figure 2B,D, respectively). In contrast, these proportions declined for authors from UMICs (figure 2C). There was little evidence of interactions between calendar year and gender (online supplemental appendix p 4p 5). Thus, although the overall proportion of female authorships increased during the study period, the probability of publishing as the first or last author did not increase substantially over time.

### Citation impact

As of May 2023, the 313 articles had received 12 272 citations (median 20, range 0–496). The articles were cited more than their peers in the co-citation network: the mean RCR was 1.92, and the median was 1.16. Two-thirds (211 articles, 67.4%) had been cited in a guideline or other clinical document. Almost all articles were openly accessible (306, 97.8%). Table 3 presents the results from the median RCR. The RCR was similar for articles with male first or last authors (p>0.50). There was some indication of a higher RCR for first authors from HICs (p=0.06) but not for last authors (p=0.48).

**Table 3:** Field-standardised citation impact by gender and affiliation of first or last author

	Relative citation ratio median (IQR)	
	Woman	Man
First author		
High income	1.3 (0.7–2.5)	1.5 (0.5–2.8)
Upper middle income	1.0 (0.4–1.9)	1.1 (0.4–1.6)
Lower income	1.0 (0.9–1.1)	1.1 (0.7–1.8)
Gender, p value	0.57	
Income, p value	0.06	
Last author		
High income	1.1 (0.7–1.9)	1.2 (0.5–2.7)
Upper middle income	1.2 (0.5–2.5)	1.4 (0.5–2.3)
Lower income	0.2 (0.2–0.2)	1.3 (0.8–1.9)
Gender, p value	0.60	
Income, p value	0.48	

P values are based on Kruskal-Wallis rank sum tests. Lower-income countries include World Bank lower middle-income countries and low-income countries.

### Analyses stratified by publication type

When restricting the analysis to the 157 regional publications, the results for gender were similar to the main analysis, with women more likely to occupy first author and men last author positions. However, in contrast to the main analysis, UMICs (mainly South Africa) contributed the largest number of authors and authorships, followed by HIC and LIC (online supplemental appendix p 6–8). UMIC and HIC (mainly South Africa and Switzerland) contributed similar numbers of first or last authorships (range 42.7%–50.3%), whereas LIC authors contributed few first (14.6%) or last authorship (3.8%). For multiregional analyses, the results for gender were also similar to the main analysis (online supplemental appendix p 9–11). Authors and authorships from HICs dominated overall and also contributed most first author (85.3%) or last author (83.2%) positions. For the other publications, there was little evidence of a gender difference in first authorships, but men again dominated the last author positions (61.0%), and authors from HICs were more common in first author (59.0%) and last author (66.1%) positions (online supplemental appendix p 12–14).

## Discussion

We analysed 313 papers authored over 13 years by 1064 researchers from a global health consortium. We found that while most articles included both male and female authors and authors from the countries contributing data, there were significant differences in authorship positions. Women were more likely to be first but less likely to be last authors compared with men. Authors from LICs or UMICs had lower chances of being first or last authors compared with those from HICs. Among the 157 articles reporting regional analyses, the UMIC and HIC (essentially South Africa and Switzerland) contributed similar numbers of first or last authorships. The proportion of women and authors from LICs increased over time, but their chances of being first or last authors did not change. Citation impact was similar for male and female authors, but slightly higher for first authors from HICs.

Strengths of this study include the large number of authors and authorships analysed and the long study period, which allowed analyses of trends over time. Standardising authorship positions by expressing them as percentiles between first and last positions allowed us to show that the proportion of female authors gradually decreased from the first to the last authorship and that there was a U-shaped relationship with country income level of authors’ affiliations. Another strength is the analysis of academic impact, based on the RCR, which is an article-based measure and more meaningful for assessing a portfolio than journal level measures such as the journal impact factor.^22^ Limitations include that the study was not planned prospectively and relevant information was not systematically available, including the level of seniority of authors, PhD student authorships or the distinction between authors’ country of origin and country of current affiliation. We could not analyse authorship according to gender identity because the use of names only allows a binary classification. Such an analysis would need to authors to self-identify their gender identity.

The findings from our collaboration, which is led by a woman from a UMIC and a man from a HIC, differ from other studies. An analysis of authors publishing in one global health journal found that, from 2013 to 2018, only a third of authors were women.^23^ In another study, of 153 articles published in 14 global health journals from January 2018 to June 2019, fewer women than men were first authors (45% compared with 55% in our study).^9^ Consistent with Rees *et al*,^8^ a review of articles published from 2014 to 2016 on health-related topics in 43 sub-Saharan African countries by Hedt-Gauthier *et al* reported that about 15% of papers had no author from the country where the study had been done, ranging from 6% for South Africa to 48% for Lesotho among the six countries participating in IeDEA-SA.^24^ The gender differences in authorship might be associated with the nature of the IeDEA-SA collaboration, which studies HIV infection in the world’s most heavily affected region. Women academics in Southern Africa might be particularly interested in studying HIV because women are disproportionately affected. The IeDEA-SA collaboration promotes the careers of younger women researchers. On the other hand, the lag in the participation of women in senior academic positions^25^ might contribute to the lower probability of women as last authors.

International research collaborations should address key aspects of social justice, namely, avoiding unequal power relations, promoting group recognition, self-development and inclusion in decision-making.^26^ The IeDEA-SA collaboration already complies with several of these and other recommendations for global health research.^26–31^ All investigators and stakeholders are encouraged to propose relevant research questions and analyses, to ensure that the study addresses research priorities relevant to the local context. The fact that many of the articles were cited in clinical guidelines indicates that IeDEA-SA research reflected such priorities. The collaboration is paired between Switzerland and South Africa through the two co-principle investigators, with other pairings within projects, including several South–South collaborations. The budget supports investigators and sites in all participating countries, including salaries, bidirectional travel and conference attendance. In recent years, the Fogarty-IeDEA Mentorship Program^32^ has strengthened the support of trainees. Roles and responsibilities are assigned in the concept sheets that propose new analyses. Finally, IeDEA researchers from HICs have repeatedly worked embedded in groups in low-income and middle-income countries.

We discussed the results at a meeting in November 2022 in South Africa, attended by over 50 collaborators from the six Southern African countries and Switzerland. The group agreed that the co-lead by researchers from South Africa and Switzerland promoted equity and excluded ‘parachutes and parasites’.^7^ Important challenges remain. Collaborators in LICs often have no academic position and lack research time. The collaboration should prioritise protected research time for those based in LICs and the mentoring of junior authors. In line with a recent consensus statement,^33 34^ the ICMJE authorship guidelines^13^ should be interpreted in an inclusive way, emphasising the ‘or’ in the first two criteria (‘substantial contributions to the conception or design of the work or the acquisition, analysis or interpretation of data for the work’ and ‘drafting the work or revising it critically for important intellectual content’). There was agreement that all authors must approve and be accountable for the final version of the paper (criteria 3 and 4). Descriptions of authors’ contributions are helpful but ‘simply scrapping authorship and move to contributorship’^35^ has not gained momentum: authorship position remains the currency for academic promotion. Joint first and last authorships were seen as a way forward while avoiding gift authorship and tokenism. There was agreement that journals should remove limits on the number of authors.

## Conclusions

Our study showed little evidence of authorship parasitism in the IeDEA-SA collaboration. Still, inequalities in authorship positions must be addressed, including inequalities by gender and the fact that researchers from LICs are ‘stuck in the middle’ on the list of authors. The IeDEA-SA consortium is committed to increasing the proportion of authors from LICs, including as first and last authors, and is currently revising its authorship guidelines. It will continue to monitor authorships and hopefully document decreasing inequalities in the coming years.

## Data Availability

The data are accessible to researchers upon reasonable request for data sharing to the corresponding authors. Requests for data need to be approved by IeDEA-SA. The code is available from https://github.com/sk-veronika/Authorships.
