# Supplementary material for: Authorship inequalities in global health research: the IeDEA Southern Africa collaboration
Source: BMJ Glob Health. 2023 Dec 15;8(12):e013316. doi: 10.1136/bmjgh-2023-013316 (PMC10729048; doi:10.1136/bmjgh-2023-013316)
Supplement: Supplementary data [file bmjgh-2023-013316supp001.pdf]

Supplement

Skrivankova et al. Authorship Inequalities in Global Health Research: the IeDEA Southern Africa Collaboration

Table 1: List of PMIDs and DOIs for all 313 articles used in the main analysis. 2

Table 2. Number of authorships by journal, in alphabetical order. 4

Table 3. Time trends: interaction between year and gender. 5

Table 4. Characteristics of authors and authorships of 157 (regional, original research) articles published by the International epidemiology Databases to Evaluate AIDS (IeDEA) in Southern Africa, 2007 to 2020. 6

Table 5. Associations of gender and country of affiliation with authorship position on 157 (regional, original research) articles published by the International epidemiology Databases to Evaluate AIDS (IeDEA) in Southern Africa, 2007 to 2020. 7

Figure 1. Proportion of Women authorships across the range of standardised authorship positions, with a weighted linear regression line (upper panel) and proportion of authorships by country income level with weighted cubic splines (lower panel). 8

Table 6. Characteristics of authors and authorships of 95 multiregional, original research articles including the International epidemiology Databases to Evaluate AIDS (IeDEA), 2007 to 2020, with participation of the Southern African region. 9

Table 7. Associations of gender and country of affiliation with authorship position on 95 multiregional, original research articles including the International epidemiology Databases to Evaluate AIDS (IeDEA), 2007 to 2020, with participation of the Southern African region. 10

Figure 2. Proportion of Women authorships across the range of standardised authorship position, with a weighted linear regression line (upper panel) and proportion of authorships by country income level with weighted cubic splines (lower panel). 11

Table 8. Characteristics of authors and authorships of 61 other publications (methods papers, commentaries, systematic reviews) acknowledging the grant of the International epidemiology Databases to Evaluate AIDS (IeDEA) in Southern Africa, 2007 to 2020. 12

Table 9. Associations of gender and country of affiliation with authorship position on 61 other publications (methods papers, commentaries, systematic reviews) acknowledging the grant of the International epidemiology Databases to Evaluate AIDS (IeDEA) in Southern Africa, 2007 to 2020. .. 13

Figure 3. Proportion of Women authorships across the range of standardised authorship position, with a weighted linear regression line (upper panel) and proportion of authorships by country income level with weighted cubic splines (lower panel). 14

Skrivankova VW, *et al. BMJ Glob Health* 2023; 8:e013316. doi: 10.1136/bmjgh-2023-013316

Table 1: List of PMIDs and DOIs for all 313 articles used in the main analysis.

PMID	*DOI*	PMID	*DOI*	PMID	*DOI*
17460471	10.1097/QAI.0b013e318042e1c3	23827024	10.5588/ijtld.13.0032	28657963	10.1097/QAD.0000000000001499
17990236	10.1086/522986	23846570	10.1097/QAI.0b013e3182a23e9a	28691434	10.7448/IAS.20.1.21552
17998240	10.1093/ije/dym216	23860481	10.1097/INF.0b013e31828c3738	28691438	10.7448/IAS.20.1.21327
18240981	10.1089/jwh.2007.0353	23873614	10.1002/sim.5912	28708808	10.1097/QAI.0000000000001499
18373510	10.1111/j.1365-3156.2008.02078.x	23874476	10.1371/journal.pone.0067956	28792285	10.1080/16549716.2017.1359923
18468529	10.1016/S0140-6736(08)60669-8	23931924	10.1016/S0140-6736(13)61713-4	28865422	10.1186/s12885-017-3549-1
18613745	10.1371/journal.pmed.0050148	24114324	10.1002/14651858.CD010309.pub2	28884524	10.7448/IAS.20.1.21947
18670668	10.2471/blt.07.044248	24147059	10.1371/journal.pone.0077697	28953328	10.7448/IAS.20.1.21902
18771599	10.1186/1471-2431-8-34	24180002	10.1097/qad.0b013e328360dbb4	29022531	10.7196/SAMJ.2017.v107i10.12362
18797643	10.2471/blt.07.045294	24260029	10.1371/journal.pmed.1001555	29112692	10.1371/journal.pmed.1002407
18981768	10.1097/QAD.0b013e3283121ca9	24267671	10.7448/IAS.16.1.18616	29143625	10.1186/s12879-017-2768-z
19053892	10.1586/14787210.6.6.785	24296645	10.3851/IMP2710	29175231	10.1016/j.jcv.2017.11.005
19142294	10.2471/blt.07.049908	24326599	10.1097/QAI.0000000000000075	29206723	10.1097/QAI.0000000000001611
19287300	10.1097/QAD.0b013e32832956ad	24363808	10.1371/journal.pone.0081037	29232366	10.1371/journal.pmed.1002468
19399157	10.1371/journal.pmed.1000066	24378936	10.1097/INF.0000000000000214	29330173	10.1136/bmjopen-2017-017405
19413745	10.1111/j.1365-3156.2009.02290.x	24378944	10.1097/INF.0000000000000222	29336004	10.1007/s10461-018-2030-8
19531928	10.1097/QAD.0b013e32832e05b2	24468999	10.1097/QAD.0000000000000143	29432229	10.1097/COH.0000000000000457
19624478	10.1111/j.1365-3156.2009.02338.x	24521144	10.1186/2046-4053-3-11	29461329	10.1097/COH.0000000000000460
19673616	10.1086/605501	24585558	10.1093/cid/ciu117	29479867	10.1002/jia2.25084
19848608	10.1086/648096	24610874	10.1093/infdis/jiu138	29508740	10.1016/S0140-6736(18)30463-X
19859538	10.1371/journal.pmed.1000173	24691205	10.1097/QAD.0000000000000135	29509590	10.1097/QAI.0000000000001667
20001527	10.1086/649007	24729433	10.1002/ijc.28894	29514233	10.1093/cid/cix1140
20003034	10.1111/j.1365-3156.2009.02445.x	24804707	10.1371/journal.pone.0096334	29517500	10.1097/COH.0000000000000461
20035163	10.1097/QAI.0b013e3181c22d19	24866364	10.1371/journal.pmed.1001650	29528851	10.1097/COH.0000000000000448
20334848	10.1016/S1473-3099(10)70026-8	24977471	10.1097/QAI.0000000000000269	29570723	10.1371/journal.pmed.1002534
20473792	10.1080/09540120903349102	25039469	10.1111/tmi.12352	29603887	10.1002/jia2.25101
20507549	10.1186/1471-2334-10-138	25117964	10.1097/QAI.0000000000000253	29682776	10.1002/sim.7654
20561308	10.1111/j.1365-3156.2010.02561.x	25196841	10.1002/ijc.29183	29727458	10.1371/journal.pmed.1002565
20588185	10.1097/QAI.0b013e3181e0c4cf	25203931	10.1371/journal.pmed.1001718	29767681	10.1093/aje/kwy093
20638120	10.1016/S0140-6736(10)60666-6	25265135	10.1097/EDE.0000000000000178	29874247	10.1371/journal.pone.0197854
20683318	10.1097/QAD.0b013e32833d45c5	25299866	10.5588/ijtld.14.0106	29881504	10.11604/pamj.2017.28.261.11300
20935554	10.1097/QAD.0b013e32833fb71f	25321185	10.1097/QAI.0000000000000399	29889240	10.1093/cid/ciy347
21085709	10.1371/journal.pone.0013899	25362193	10.1093/infdis/jiu601	29912371	10.1093/cid/ciy304
21106512	10.1136/sti.2010.044255	25392857	10.1097/qad.0000000000000446	29956882	10.1002/jia2.25149
21107266	10.1097/QAI.0b013e3182060610	25393941	10.1097/QAI.0000000000000360	29972632	10.1002/jia2.25157
21109173	10.1016/S1473-3099(10)70280-2	25399412	10.1093/aje/kwu295	29991395	10.5588/ijtld.17.0766
21152392	10.1371/journal.pone.0014149	25501345	10.1097/QAI.0000000000000380	29994916	10.1097/QAI.0000000000001745
21267057	10.1371/journal.pmed.1000390	25516092	10.7448/IAS.17.1.19045	30005013	10.1097/QAD.0000000000001942
21272350	10.1186/1471-2334-11-31	25562494	10.1097/QAD.0000000000000506	30019488	10.1002/hep.30183
21317578	10.1097/QAI.0b013e318202c40e	25701987	10.1016/S2214-109X(14)70383-3	30063542	10.1097/EDE.0000000000000905
21418449	10.1111/j.1365-3156.2011.02767.x	25793531	10.1371/journal.pone.0119299	30215763	10.1093/jpids/piy087
21436714	10.1097/QAI.0b013e3182199ee9	25859596	10.1093/ije/dyu271	30308013	10.1371/journal.pone.0205455
21593078	10.1093/ije/dyr080	25884599	10.1186/s12885-015-1171-7	30452634	10.1093/cid/ciy960
21681057	10.1097/QAD.0b013e328349822f	25950205	10.1097/QAI.0000000000000669	30531296	10.1097/QAI.0000000000001923
21756512	10.5588/ijtld.10.0249	26112433	10.1016/j.jclinepi.2015.04.002	30548817	10.1002/jia2.25215
21834797	10.1111/j.1365-3156.2011.02854.x	26167620	10.1097/QAI.0000000000000683	30549445	10.1002/jia2.25202
21935448	10.1371/journal.pone.0024737	26175054	10.1002/ijc.29687	30614622	10.1002/jia2.25200
21957868	10.1186/1471-2334-11-258	26192393	10.1097/INF.0000000000000801	30657644	10.1002/jia2.25218
21969875	10.1371/journal.pone.0025244	26214336	10.1097/EDE.0000000000000334	30681241	10.1111/tmi.13209
22016763	10.1371/journal.pone.0025098	26218412	10.1097/QAI.0000000000000773	30718257	10.1128/AAC.02175-18
22039357	10.1371/journal.pmed.1001111	26288079	10.1371/journal.pone.0135179	30744962	10.1016/S1473-3099(18)30673-X
22067665	10.1097/QAI.0b013e31823edb6a	26356987	10.1016/j.jcv.2015.08.019	30882732	10.1097/INF.0000000000002156
22089376	10.1097/QAD.0b013e32834e1b5f	26375465	10.1097/QAI.0000000000000691	30891866	10.1111/tmi.13233
22095194	10.1097/QAD.0b013e32834ed814	26379169	10.1097/INF.0000000000000912	30950882	10.1097/QAD.0000000000002221
22103326	10.1111/j.1468-1293.2011.00947.x	26407355	10.1093/jpids/piu020	31148372	10.1002/jia2.25304
22114687	10.1371/journal.pone.0027763	26411694	10.7448/IAS.18.1.20240	31181056	10.1371/journal.pmed.1002822
22116153	10.1128/JCM.05392-11	26423252	10.1016/S2352-3018(15)00087-9	31215037	10.1002/ijc.32260

22205933	10.1371/journal.pone.0027919	26423550	10.1016/S2352-3018(15)00113-7	31233494	10.1371/journal.pcbi.1007083
22289654	10.1186/1758-2652-15-5	26470034	10.1097/QAI.0000000000000748	31242142	10.1097/QAI.0000000000002060
22421243	10.1097/QAD.0b013e3283536988	26479876	10.1097/EDE.0000000000000412	31332437	10.1093/infdis/jiz362
22433846	10.1097/QAI.0b013e3182557785	26685125	10.7448/IAS.18.1.20628	31436859	10.1002/sim.8340
22470121	10.1128/AAC.06460-11	26691682	10.1097/QAD.0000000000001009	31507083	10.1002/jia2.25392
22479556	10.1371/journal.pone.0034186	26700300	10.1136/jech-2015-206629	31613956	10.1093/infdis/jiz450
22668950	10.7196/samj.5625	26741583	10.1097/INF.0000000000000926	31623428	10.1002/jia2.25331
22695302	10.1097/QAD.0b013e3283565dd1	26852390	10.1186/s12885-016-2080-0	31682261	10.1093/infdis/jiz547
22742573	10.1186/1471-2334-12-147	26906162	10.1097/INF.0000000000001117	31698524	10.1111/tmi.13334
22743595	10.1097/QAI.0b013e3182632540	26939736	10.1016/S2352-3018(16)00016-3	31702088	10.1002/jia2.25412
22762421	10.5588/ijtld.12.0364	26945749	10.1186/s13104-016-1943-4	31725119	10.1097/INF.0000000000002516
22768066	10.1371/journal.pone.0039246	26972514	10.1016/j.epidem.2015.10.001	31725750	10.1371/journal.pone.0224837
22808948	10.1111/j.1365-3156.2012.03041.x	27032097	10.1371/journal.pone.0152043	31800405	10.1097/QAD.0000000000002358
22845473	10.1586/erm.12.52	27036993	10.1016/S2352-3018(16)00008-4	31805028	10.1097/QAD.0000000000002357
22874482	10.1097/QAD.0b013e3283565ebe	27049853	10.1371/journal.pone.0151762	31814312	10.1002/jia2.25423
22879816	10.1371/journal.pmed.1001281	27062038	10.1002/ijc.30129	31900473	10.1093/cid/ciz1224
22948267	10.1097/QAD.0b013e328359ab0c	27064254	10.1038/ng.3548	31997583	10.1002/jia2.25441
22973181	10.1371/journal.pmed.1001304	27073928	10.1371/journal.pone.0153243	32003159	10.1002/jia2.25447
22974345	10.1111/j.1365-3156.2012.03073.x	27098265	10.1002/ijc.30154	32032304	10.1097/QAI.0000000000002313
22987878	10.1542/peds.2011-3020	27254748	10.1097/COH.0000000000000298	32119663	10.1371/journal.pone.0228601
22994151	10.1111/j.1365-3156.2012.03089.x	27282427	10.1007/s40264-016-0434-9	32219384	10.1093/aje/kwaa049
23172344	10.1136/sextrans-2012-050658	27293220	10.7448/IAS.19.1.20711	32357149	10.1371/journal.pone.0220165
23187940	10.1097/QAI.0b013e31827b70bf	27342220	10.1093/ije/dyw097	32406983	10.1002/jia2.25476
23199369	10.1111/tmi.12026	27428744	10.1097/QAD.0000000000001197	32414825	10.1136/bmjopen-2019-035246
23252488	10.2217/fmb.12.128	27461920	10.1093/cid/ciw500	32433224	10.1097/INF.0000000000002680
23300635	10.1371/journal.pone.0052297	27499385	10.1111/tmi.12764	32448379	10.1186/s13104-020-05081-7
23344547	10.1097/QAI.0b013e318287c1fe	27578823	10.1093/cid/ciw519	32469985	10.1371/journal.pone.0233693
23364513	10.1097/QAI.0b013e318288b39d	27596685	10.1016/j.ijid.2016.08.028	32589346	10.1002/jia2.25517
23418463	10.1371/journal.pone.0055824	27685986	10.1097/COH.0000000000000331	32589347	10.1002/jia2.25510
23418518	10.1371/journal.pone.0056088	27716730	10.1097/COH.0000000000000303	32589367	10.1002/jia2.25530
23437135	10.1371/journal.pone.0056446	27776039	10.1097/QAD.0000000000001307	32603374	10.1371/journal.pone.0235471
23457534	10.1371/journal.pone.0056248	27798628	10.1038/ng.3704	32640106	10.1002/jia2.25546
23460854	10.1371/journal.pone.0057422	28077521	10.1093/cid/ciw880	32718966	10.1128/AAC.00782-20
23462219	10.1097/QAD.0b013e328360a4e5	28092681	10.1038/ng.3767	32722897	10.1002/jia2.25580
23469035	10.1371/journal.pone.0057611	28102610	10.1111/tmi.12843	32762660	10.1186/s12913-020-05517-7
23472118	10.1371/journal.pone.0057907	28158504	10.1093/cid/cix122	32828836	10.1016/j.jclinepi.2020.08.012
23498041	10.7196/samj.6024	28192529	10.1371/journal.pone.0169762	32847906	10.1136/bmjopen-2019-036214
23505379	10.1371/journal.pgen.1003318	28234689	10.1097/QAI.0000000000001335	32853246	10.1371/journal.pone.0237772
23572010	10.1097/QAI.0b013e318293ae48	28296798	10.1097/QAD.0000000000001321	32868333	10.1128/AAC.00513-20
23575330	10.5588/ijtld.12.0811	28364561	10.7448/IAS.20.1.20933	32910063	10.1097/QAD.0000000000002683
23585736	10.1371/journal.pmed.1001418	28369607	10.1093/molbev/msx107	32971055	10.1016/S2214-109X(20)30279-5
23614996	10.1097/QAI.0b013e3182969cc1	28440019	10.1002/ijc.30749	33034417	10.1002/jia2.25617
23633367	10.1002/14651858.CD009153.pub3	28482962	10.5588/ijtld.16.0843	33198721	10.1186/s12905-020-01121-3
23660577	10.1097/QAD.0b013e328362d887	28510998	10.1111/tmi.12896	33283916	10.1002/jia2.25644
23674929	10.1371/journal.pmed.1001441	28520616	10.1097/QAI.0000000000001453	33315863	10.1371/journal.pmed.1003397
23751258	10.1097/QAD.0b013e328363bf7f	28530036	10.7448/IAS.20.4.21520	33371015	10.1136/bmjopen-2020-037955
23755122	10.1371/journal.pone.0064392	28530037	10.7448/IAS.20.4.21668		
23774112	10.1016/j.jclinepi.2013.03.013	28530039	10.7448/IAS.20.4.21528		

Table 2. Number of authorships by journal, in alphabetical order.

Journal	No. of authorships	%
AIDS (London, England)	329	9.62
AIDS and behavior	6	0.18
AIDS care	17	0.50
American journal of epidemiology	23	0.67
Antimicrobial agents and chemotherapy	72	2.10
Antiviral therapy	13	0.38
BMC Health Services Research	9	0.26
BMC cancer	48	1.40
BMC infectious diseases	65	1.90
BMC pediatrics	5	0.15
BMC research notes	28	0.82
BMC womens health	6	0.18
BMJ open	37	1.08
Bulletin of the World Health Organiza..	35	1.02
Clinical infectious diseases : an off..	156	4.56
Current opinion in HIV and AIDS	21	0.61
Current opinion in infectious diseases	13	0.38
Drug safety	15	0.44
Epidemics	9	0.26
Epidemiology (Cambridge, Mass.)	31	0.91
Expert review of anti-infective therapy	1	0.03
Expert review of molecular diagnostics	2	0.06
Future microbiology	2	0.06
Global health action	9	0.26
HIV medicine	10	0.29
Hepatology	5	0.15
International journal of cancer	83	2.43
International journal of epidemiology	80	2.34
International journal of infectious d..	14	0.41
Journal of acquired immune deficiency..	397	11.60
Journal of clinical epidemiology	26	0.76
Journal of clinical microbiology	20	0.58
Journal of clinical virology : the of..	19	0.56
Journal of epidemiology and community..	10	0.29
Journal of the International AIDS Soc..	415	12.13
Journal of the Pediatric Infectious D..	33	0.96
Journal of women's health (2002)	15	0.44
Lancet (London, England)	19	0.56
Molecular biology and evolution	15	0.44
Nature genetics	139	4.06
PLoS computational biology	8	0.23
PLoS genetic	23	0.67
PLoS medicine	219	6.40
PLoS one	323	9.44
Pediatrics	16	0.47
Sexually transmitted infections	16	0.47
South African medical journal = Suid-..	17	0.50

Statistics in medicine	11	0.32
Systematic reviews	8	0.23
The Cochrane database of systematic r..	11	0.32
The Journal of infectious diseases	56	1.64
The Lancet. Global health	15	0.44
The Lancet. Infectious diseases	32	0.94
The Pan African medical journal	8	0.23
The Pediatric infectious disease jour..	133	3.89
The international journal of tubercul..	68	1.99
The Lancet. HIV	46	1.34
Tropical medicine & international hea..	159	4.65

Table 3. Time trends: interaction between year and gender.

Comparison / Authorship position	***Multivariable models: Interaction with year***	
	OR (95%CI)	***p-value***
Year*Gender: Women vs Men		
0 percentile (first)	1.03 (0.93 - 1.14)	0.6
1–33 percentile	1.08 (1.00 - 1.17)	0.05
34–66 percentile	1	
67–99 percentile	1.02 (0.95 - 1.10)	0.52
100 percentile (last)	1.07 (0.97 - 1.19)	0.16

Results from generalized multinomial regression models (GMRM) with random intercept for authors. Multivariable models adjusted for gender and income level of the country of affiliation, and additionally including an interaction between year and gender (model 1) or year and affiliation (model 2).

Table 4. Characteristics of authors and authorships of 157 (regional, original research) articles published by the International epidemiology Databases to Evaluate AIDS (IeDEA) in Southern Africa, 2007 to 2020.

	Authors# N (%)	*Authorships N (%)*	*No. of articles /author Mean (SD)*	*First authorships N (%)*	*Last authorships N (%)*	*First and/or last authorships N (%)*
Total	461	1535	3.3 (7.1)	157	157	157
Gender						
Women	239 (51.8%)	630 (41.0%)	2.6 (5.4)	84 (53.5%)	59 (37.6%)	109 (69.4%)
Men	222 (48.2%)	905 (59.0%)	4.1 (8.4)	73 (46.5%)	98 (62.4%)	123 (78.3%)
Country of affiliation						
High income	174 (37.7%)	486 (31.7%)	3.4 (8.3)	67 (42.7%)	72 (45.9%)	95 (60.5%)
Women	87 (50.0%)	188 (38.7%)	2.3 (5.1)	36 (53.7%)	36 (50.0%)	59 (62.1%)
Men	87 (50.0%)	298 (61.3%)	4.6 (10)	31 (46.3%)	36 (50.0%)	56 (58.9%)
Upper-middle income	184 (39.9%)	799 (52.1%)	3.9 (7.4)	67 (42.7%)	79 (50.3%)	89 (56.7%)
Women	116 (63.0%)	385 (48.2%)	3.3 (6.3)	44 (65.7%)	23 (29.1%)	56 (62.9%)
Men	68 (37.0%)	414 (51.8%)	4.9 (9.0)	23 (34.3%)	56 (70.9%)	66 (74.2%)
Lower income	103 (22.3%)	250 (16.3%)	2.2 (2.9)	23 (14.6%)	6 (3.8%)	24 (15.3%)
Women	36 (35.0%)	57 (22.8%)	1.3 (0.8)	4 (17.4%)	0 (0%)	4 (16.7%)
Men	67 (65.0%)	193 (77.2%)	2.7 (3.5)	19 (82.6%)	6 (100%)	23 (95.8%)

Lower income includes World Bank lower-middle income countries and low-income countries.

For authors who listed multiple affiliations, we defined the main affiliation as the place where the author spent most of their time when the article was published.

The percentage of Women/Men within an affiliation income category were calculated from total in the corresponding income category.

Skrivankova VW, *et al. BMJ Glob Health* 2023; 8:e013316. doi: 10.1136/bmjgh-2023-013316

Table 5. Associations of gender and country of affiliation with authorship position on 157 (regional, original research) articles published by the International epidemiology Databases to Evaluate AIDS (IeDEA) in Southern Africa, 2007 to 2020.

Comparison / Authorship position	***Univariable models***		***Multivariable model***	
	OR (95%CI)	***p-value***	***OR (95%CI)***	***p-value***
Gender: Women vs Men				
0 percentile (first)	1.98 (0.98 – 4.00)	0.06	2.05 (0.99 - 4.24)	0.05
1–33	1.13 (0.64 - 1.96)	0.68	1.12 (0.57 - 2.21)	0.75
34–66	1		1	
67–99	0.84 (0.49 - 1.46)	0.54	0.82 (0.45 - 1.49)	0.52
100 percentile (last)	0.69 (0.37 - 1.30)	0.25	0.62 (0.31 - 1.23)	0.17
Country of affiliation				
Lower vs high income				
0 percentile (first)	0.31 (0.12 - 0.79)	0.01	0.36 (0.14 - 0.93)	0.03
1–33	0.92 (0.40 - 2.10)	0.85	0.95 (0.41 - 2.22)	0.91
34–66	1		1	
67–99	0.60 (0.29 - 1.24)	0.17	0.60 (0.28 - 1.27)	0.18
100 percentile (last)	0.15 (0.04 - 0.54)	<0.001	0.14 (0.04 - 0.54)	<0.001
Upper-middle vs high income				
0 percentile (first)	0.39 (0.18 - 0.83)	0.01	0.36 (0.17 - 0.79)	0.01
1–33	1.06 (0.52 - 2.14)	0.87	1.07 (0.52 - 2.20)	0.86
34–66	1		1	
67–99	0.71 (0.38 - 1.33)	0.29	0.74 (0.39 - 1.42)	0.37
100 percentile (last)	0.51 (0.26 - 0.99)	0.05	0.53 (0.27 - 1.06)	0.07

Results from generalized multinomial regression model (GMRM) with random intercept for authors. Multivariable model is adjusted for gender and income level of the country of affiliation. Lower income includes World Bank lower-middle income countries and low-income countries.

Skrivankova VW, *et al. BMJ Glob Health* 2023; 8:e013316. doi: 10.1136/bmjgh-2023-013316

Figure 1. Proportion of Women authorships across the range of standardised authorship positions, with a weighted linear regression line (upper panel) and proportion of authorships by country income level with weighted cubic splines (lower panel).

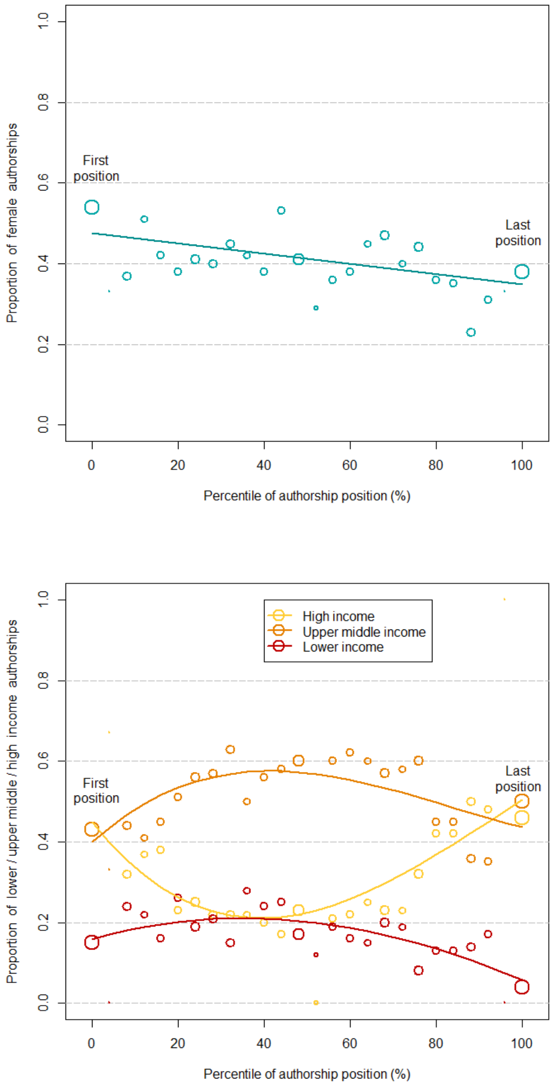

Based on 157 (regional, original research) articles published by the International epidemiology Databases to Evaluate AIDS (IeDEA) in Southern Africa, 2007 to 2020. Lower income includes World Bank lower-middle income countries and low-income countries. The size of the circles is proportional to the number of authorships in each position.

Table 6. Characteristics of authors and authorships of 95 multiregional, original research articles including the International epidemiology Databases to Evaluate AIDS (IeDEA), 2007 to 2020, with participation of the Southern African region.

	Authors[#] N (%)	Authorships N (%)	No. of articles /author Mean (SD)	First authorships N (%)	Last authorships N (%)	First and/or last authorships N (%)
**Total**	613	1484	2.4 (3.9)	95	95	95
**Gender**						
**Women**	306 (49.9%)	666 (44.9%)	2.2 (3.1)	58 (61.1%)	22 (23.2%)	67 (70.5%)
**Men**	307 (50.1%)	818 (55.1%)	2.7 (4.6)	37 (38.9%)	73 (76.8%)	82 (86.3%)
**Country of affiliation**						
**High income**	357 (58.2%)	889 (59.9%)	2.7 (4.6)	81 (85.3%)	79 (83.2%)	88 (92.6%)
Women	177 (49.6%)	414 (46.6%)	2.4 (3.3)	53 (65.4%)	14 (17.7%)	59 (67.0%)
Men	180 (50.4%)	475 (53.4%)	2.9 (5.6)	28 (34.6%)	65 (82.3%)	72 (81.8%)
**Upper-middle income**	119 (19.4%)	310 (20.9%)	2.4 (3.6)	11 (11.6%)	13 (13.7%)	17 (17.9%)
Women	73 (61.3%)	152 (49%)	2.0 (3.6)	5 (45.5%)	7 (53.8%)	10 (58.8%)
Men	46 (38.7%)	158 (51%)	2.9 (3.6)	6 (54.5%)	6 (46.2%)	12 (70.6%)
**Lower income**	137 (22.3%)	285 (19.2%)	1.8 (1.5)	3 (3.2%)	3 (3.2%)	6 (6.3%)
Women	56 (40.9%)	100 (35.1%)	1.6 (1.0)	0 (0%)	1 (33.3%)	1 (16.7%)
Men	81 (59.1%)	185 (64.9%)	2 (1.8)	3 (100%)	2 (66.7%)	5 (83.3%)

Lower income includes World Bank lower-middle income countries and low-income countries.
For authors who listed multiple affiliations, we defined the main affiliation as the place where the author spent most of their time when the article was published.
The percentage of Women/Men within an affiliation income category were calculated from total in the corresponding income category.

Skrivankova VW, *et al. BMJ Glob Health* 2023; 8:e013316. doi: 10.1136/bmjgh-2023-013316

Table 7. Associations of gender and country of affiliation with authorship position on 95 multiregional, original research articles including the International epidemiology Databases to Evaluate AIDS (IeDEA), 2007 to 2020, with participation of the Southern African region.

Comparison / Authorship position	***Univariable models***		***Multivariable model***	
	OR (95%CI)	***p-value***	***OR (95%CI)***	***p-value***
Gender: Women vs Men				
0 percentile (first)	2.08 (1.06 - 4.08)	0.03	1.97 (1.00 - 3.90)	0.05
1–33	1.12 (0.63 - 1.99)	0.71	1.13 (0.73 - 1.74)	0.59
34–66	1		1	
67–99	1.05 (0.62 - 1.75)	0.87	1.02 (0.67 - 1.58)	0.91
100 percentile (last)	0.52 (0.27 - 10)	0.05	0.49 (0.23 - 1.08)	0.08
Country of affiliation				
Lower vs high income				
0 percentile (first)	0.06 (0.02 - 0.24)	<0.001	0.07 (0.02 - 0.27)	<0.001
1–33	0.45 (0.23 - 0.87)	0.02	0.46 (0.28 - 0.76)	<0.001
34–66	1		1	
67–99	0.51 (0.29 - 0.90)	0.02	0.51 (0.31 - 0.84)	0.01
100 percentile (last)	0.11 (0.03 - 0.39)	<0.001	0.11 (0.03 - 0.39)	<0.001
Upper-middle vs high income				
0 percentile (first)	0.19 (0.07 - 0.50)	<0.001	0.17 (0.07 - 0.44)	<0.001
1–33	0.42 (0.19 - 0.90)	0.03	0.42 (0.24 - 0.72)	<0.001
34–66	1		1	
67–99	0.70 (0.37 - 1.32)	0.27	0.67 (0.39 - 1.14)	0.14
100 percentile (last)	0.34 (0.15 - 0.81)	0.01	0.29 (0.11 - 0.80)	0.02

Results from generalized multinomial regression model (GMRM) with random intercept for authors. Multivariable model is adjusted for gender and income level of the country of affiliation. Lower income includes World Bank lower-middle income countries and low-income countries.

Skrivankova VW, *et al. BMJ Glob Health* 2023; 8:e013316. doi: 10.1136/bmjgh-2023-013316

Figure 2. **Proportion of Women authorships across the range of standardised authorship position, with a weighted linear regression line (upper panel) and proportion of authorships by country income level with weighted cubic splines (lower panel).**

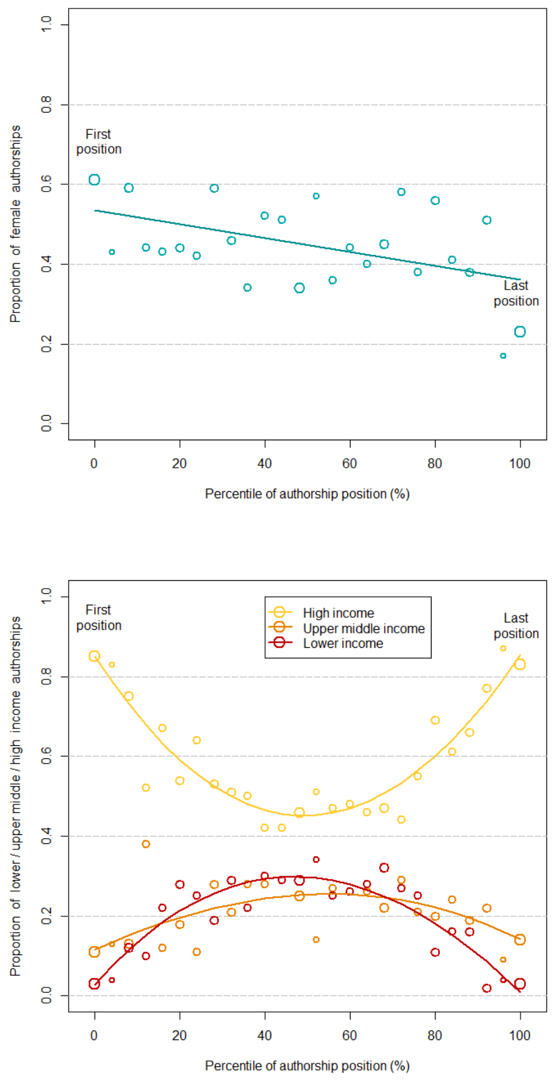

Based on 95 (multiregional, original research) articles including the International epidemiology Databases to Evaluate AIDS (IeDEA) in Southern Africa, 2007 to 2020. Lower income includes World Bank lower-middle income countries and low-income countries. The size of the circles is proportional to the number of authorships in each position.

Skrivankova VW, *et al. BMJ Glob Health* 2023; 8:e013316. doi: 10.1136/bmjgh-2023-013316

Table 8. Characteristics of authors and authorships of 61 other publications (methods papers, commentaries, systematic reviews) acknowledging the grant of the International epidemiology Databases to Evaluate AIDS (IeDEA) in Southern Africa, 2007 to 2020.

	Authors[#] N (%)	*Authorships N (%)*	*No. of articles /author Mean (SD)*	*First authorships N (%)*	*Last authorships N (%)*	*First and/or last authorships N (%)*
Total	206	402	2 (2.8)	61	59*	61
Gender						
Women	108 (52.4%)	184 (45.8%)	1.7 (2.0)	31 (50.8%)	23 (39%)	37 (60.7%)
Men	98 (47.6%)	218 (54.2%)	2.2 (3.5)	30 (49.2%)	36 (61%)	43 (70.5%)
Country of affiliation						
High income	151 (73.3%)	304 (75.6%)	2.1 (3.2)	36 (59%)	39 (66.1%)	45 (73.8%)
Women	77 (51%)	129 (42.4%)	1.9 (2.3)	15 (41.7%)	17 (43.6%)	23 (51.1%)
Men	74 (49%)	175 (57.6%)	2.4 (3.9)	21 (58.3%)	22 (56.4%)	29 (64.4%)
Upper-middle income	36 (17.5%)	78 (19.4%)	1.7 (1.6)	22 (36.1%)	20 (33.9%)	27 (44.3%)
Women	24 (66.7%)	48 (61.5%)	1.3 (1.2)	14 (63.6%)	6 (30.0%)	17 (63.0%)
Men	12 (33.3%)	30 (38.5%)	2.4 (2.1)	8 (36.4%)	14 (70.0%)	15 (55.6%)
Lower income	19 (9.2%)	20 (5.0%)	1.1 (0.2)	3 (4.9%)	0 (0%)	3 (4.9%)
Women	7 (36.8%)	7 (35.0%)	1.0 (0.0)	2 (66.7%)	0 (0%)	2 (66.7%)
Men	12 (63.2%)	13 (65.0%)	1.1 (0.3)	1 (33.3%)	0 (0%)	1 (33.3%)

* Authorships from two single-author publications were treated as first authorships.
Lower-income includes World Bank lower-middle income countries and low-income countries.
For authors who listed multiple affiliations, we defined the main affiliation as the place where the author spent most of their time when the article was published.
The percentage of Women/Men within an affiliation income category were calculated from total in the corresponding income category.

Skrivankova VW, *et al. BMJ Glob Health* 2023; 8:e013316. doi: 10.1136/bmjgh-2023-013316

Table 9. Associations of gender and country of affiliation with authorship position on 61 other publications (methods papers, commentaries, systematic reviews) acknowledging the grant of the International epidemiology Databases to Evaluate AIDS (IeDEA) in Southern Africa, 2007 to 2020.

Comparison / Authorship position	***Univariable models***		***Multivariable model***	
	OR (95%CI)	***p-value***	***OR (95%CI)***	***p-value***
Gender: Women vs Men				
0 percentile (first)	1.26 (0.43 - 3.70)	0.67	1.22 (0.40 - 3.74)	0.73
1–33	1.65 (0.61 - 4.49)	0.33	1.72 (0.62 - 4.75)	0.29
34–66	1		1	
67–99	1.31 (0.52 - 3.34)	0.57	1.40 (0.54 - 3.65)	0.49
100 percentile (last)	0.97 (0.38 - 2.46)	0.95	0.89 (0.35 - 2.28)	0.82
Country of affiliation				
Lower vs high income				
0 percentile (first)	1.88 (0.23 - 15.6)	0.56	1.94 (0.24 - 15.8)	0.53
1–33	1.09 (0.16 - 7.59)	0.93	1.18 (0.17 - 8.22)	0.87
34–66	1		1	
67–99	1.80 (0.31 - 10.3)	0.51	1.90 (0.33 - 10.8)	0.47
100 percentile (last)	0.00 (NA)	1.00	0.00 (NA)	1.00
Upper-middle vs high income				
0 percentile (first)	3.45 (0.90 - 13.2)	0.07	3.35 (0.87 - 12.9)	0.08
1–33	0.77 (0.19 - 3.11)	0.72	0.70 (0.17 - 2.87)	0.62
34–66	1		1	
67–99	0.92 (0.25 - 3.32)	0.90	0.88 (0.24 - 3.18)	0.84
100 percentile (last)	3.68 (1.25 - 10.8)	0.02	3.72 (1.26 – 11.0)	0.02

Results from generalized multinomial regression model (GMRM) with random intercept for authors. Multivariable model is adjusted for gender and income level of the country of affiliation. Lower income includes World Bank lower-middle income countries and low-income countries.

Skrivankova VW, *et al. BMJ Glob Health* 2023; 8:e013316. doi: 10.1136/bmjgh-2023-013316

***Figure 3*. Proportion of Women authorships across the range of standardised authorship position, with a weighted linear regression line (upper panel) and proportion of authorships by country income level with weighted cubic splines (lower panel).**

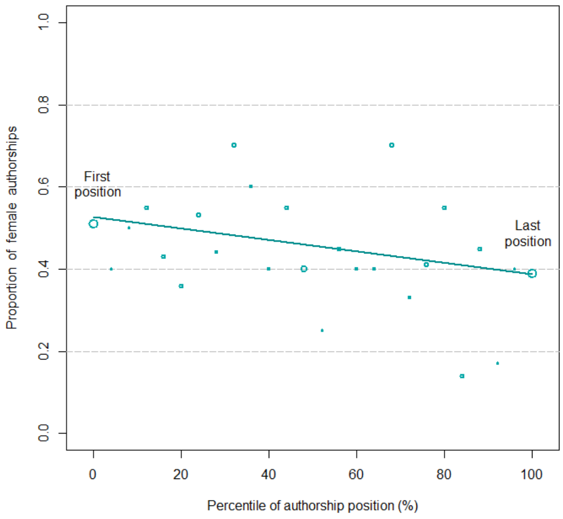

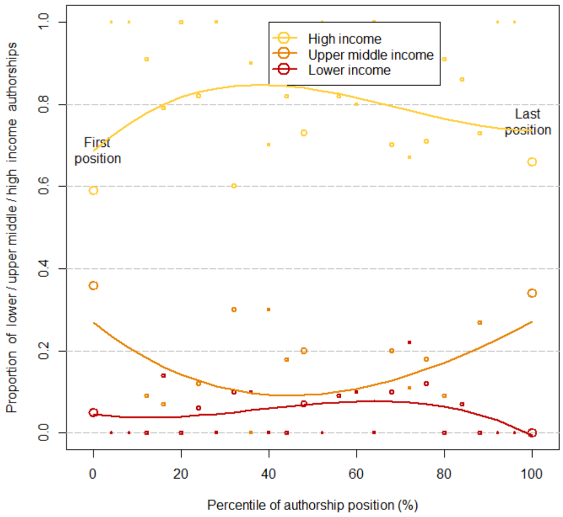

Based on 61 other publications (methods papers, commentaries, systematic reviews) acknowledging the grant of the International epidemiology Databases to Evaluate AIDS (IeDEA) in Southern Africa, 2007 to 2020. Lower income includes World Bank lower-middle income countries and low-income countries. The size of the circles is proportional to the number of authorships in each position.
